# UV-B Induced Changes in the Secondary Metabolites of *Morus alba* L. Leaves

**DOI:** 10.3390/molecules15052980

**Published:** 2010-04-27

**Authors:** Xi-Da Gu, Ming-Yao Sun, Lin Zhang, Hong-Wei Fu, Lei Cui, Run-Ze Chen, Da-Wei Zhang, Jing-Kui Tian

**Affiliations:** 1 The Key Laboratory of Biomedical Engineering Ministry of Education, Department of Biomedical Engineering, Zhejiang University, Hangzhou 310027, Zhejiang, China; E-Mails: guxida@zju.edu.cn (X.-D.G.); sunmingyao.360@163.com (M.-Y.S.); zhanglin@zju.edu.cn (L.Z.); fhw668@zju.edu.cn (H.-W.F.); cuileibox@163.com (L.C); chenrunze_2008@163.com (R.-Z.C); 2 College of Pharmaceutical Sciences, Zhejiang University, Hangzhou 310058, Zhejiang, China; 3 Department of Biological Sciences, University of Wisconsin—Milwaukee, Milwaukee, WI 53211, USA

**Keywords:** UV radiation, chalcomoracin, moracin N, Diels-Alder type adducts, mulberry leaves

## Abstract

Ultraviolet-B (UV-B) radiation is harmful to plants and human beings. Many secondary metabolites, like flavonoids, alkaloids, and lignin, are UV-B absorbing compounds, which can protect the genetic material of plants. Furthermore, they are active components of herbal drugs. UV-B radiation can activate the self-protective secondary metabolism system. The results of this paper provide a method to induce bioactive secondary metabolites from mulberry leaves (*Morus alba* L.) by UV-B irradiation *in vitro*. Five significantly different chromatographic peaks were found by HPLC fingerprint after induction, from which two active compounds were identified: One was chalcomoracin, a natural Diels-Alder type adduct with antibacterial activity; the other one was moracin N, which is a precursor of chalcomoracin. Their contents were 0.818 mg/g and 0.352 mg/g by dry weight, respectively.

## 1. Introduction

The notion that anthropogenic emissions might deplete stratospheric ozone, leading to greater atmospheric transmission of ultraviolet-B radiation (UV-B: 280 to 320 nm) and higher surface fluxes, emerged in the early 1970s, with a focus on nitrogen oxide emissions from high-altitude aircraft [1]. UV-B radiation has many direct and indirect effects on medical plants, including damages to DNA, proteins and membranes, alterations in transpiration and photosynthesis, and changes in growth, development and morphology. According to some studies [2], UV-B exposure resulted in a reduction in biomass accumulation.

Light-absorbing phenolic compounds, as a group of phenylalanine-derived aromatic secondary products, have been implicated in protecting plants from the damaging effects of UV-B radiation [3]. The antioxidant capacity of these compounds can selectively resist the free radicals generated by UV-B irradiation [4,5]. In order to deal with the increasing solar ultraviolet-B radiation, researchers in the field of plant biology and environmental science have focused on UV-B stress physiology, especially as it affects agricultural yields. In the process of searching for a UV-B-proof solution, some interesting phenomena have been discovered, as follows:

After being irradiated for 45 days, the total content of phenolic compounds in a tea callus culture grown under supplementary UV-B irradiation was almost 1.5 times higher than in a control culture [4]; HPLC results indicated that *Valencia orange* contained a trace amount (0.36 mg/g) of scoparone in untreated fruit, while the concentration of scoparone increased in UV-irradiated fruit (15.2 mg·g^-1^) [5]. The effectiveness of UV-B irradiation not only increases the production of secondary metabolites, but also produces new compounds. Through the research of phenolic acids in UV-B irradiated rice, two new compounds were separated and identified: Isoorientin-2′′-O-*β*-[6-O-E-*p*-coumaroyl-glucopyranoside] and isoorientin-2′′-O-*β*-[6-O-E-feruloylglucopyranoside] [6]. UV-B treated *Brassica oleracea* var. botrytis contains three new alkaloids: caulilexins A-C. Caulilexin A exhibited considerable anti-fungus activity against *R. solani* and *S. sclerotiorum* (MICs: 0.5 mM and 0.1 mM) [7]. 

In our experiment, a UV-B inducing method was established to stimulate mulberry leaves’ secondary metabolism *in vitro*. Mulberry leaves have long been used in Chinese medicine for the prevention and treatment of diabetes, as they contain active compounds which can suppress high blood sugar levels. Mulberry leaves significantly reduced fasting blood glucose levels in the diabetic bodies; they also lowered cholesterol levels in human bodies [10,11,12,13,14].

Mulberry leaves contain a lot of active secondary metabolites, like flavonoids, alkaloids, and phenylpropanoids. The theory of natural product chemistry indicates that a majority of UV-absorbing substances are active molecules produced by the secondary metabolism of plants. In other words, UV-B irradiation could be utilized to promote the quality of medical plants, or even to induce some rare compounds. Plant’s UV-B stress physiology has been a focus of botany and agronomy for a long time, but related research has not been reported in the area of natural medicine. Since UV-B irradiation is harmful to the genetic material of plants [15], it is hard to design a UV-B inducing method *in vivo*. The objectives of the experiments presented here were: (i) set up a UV-B inducing method of mulberry leaves *in vitro*; (ii) extract, isolate, and identify the induced products; (iii) semi-quantitative analysis the induced compounds by HPLC. 

After the processes of radiation, extraction, and separation, five significantly changed chromatographic peaks were found by HPLC fingerprinting, and two of them were identified. One was chalcomoracin, a natural Diels-Alder type adduct; the other one was moracin N, which is the precursor of chalcomoracin. Their contents were 0.818 mg/g and 0.352 mg/g by dry weight, respectively. Chalcomoracin exhibited considerable antimicrobial activity against methicillin-sensitive *Staphylococcus aureus* (MSSAs, strains FDA 209P and Smith) and methicillin-resistant *Staphylococcus aureus* (MRSAs, strains K3 and ST28) [16].

## 2. Results

### 2.1. Screening Condition of Induction

**Figure 1:**
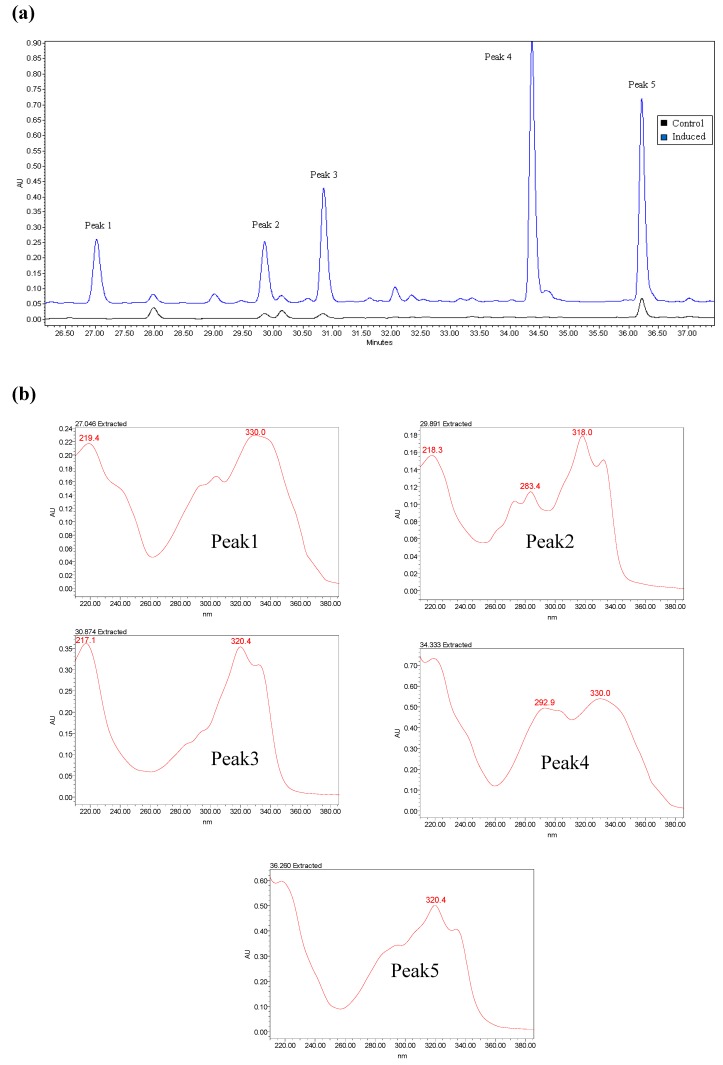
(a) The analyses of sample solutions in HPLC fingerprint. HPLC chromatograms of the control/induced mulberry leaves’ extract. The blue curve representsthe induced sample, and the black curve represents the control sample. A 250 mm WatersSymmetry C_18_ column was utilized for this experiment. (b) Spectrogram of each target compound. Five significantly different chromatographic peaks were found by HPLC fingerprint after induction.

In the preliminary experiment, mock-treated and treated samples were compared by HPLC-DAD fingerprinting (Figure 1a). Five significantly changed chromatographic peaks were found. These peaks, all of which had strong absorptions at 310-330 nm, were chosen as target compounds (Figure 1b). The peak area (v_*_s) was selected as indicator for condition screening.

#### 2.1.1. Screening the Month of Induction

**Figure 2:**
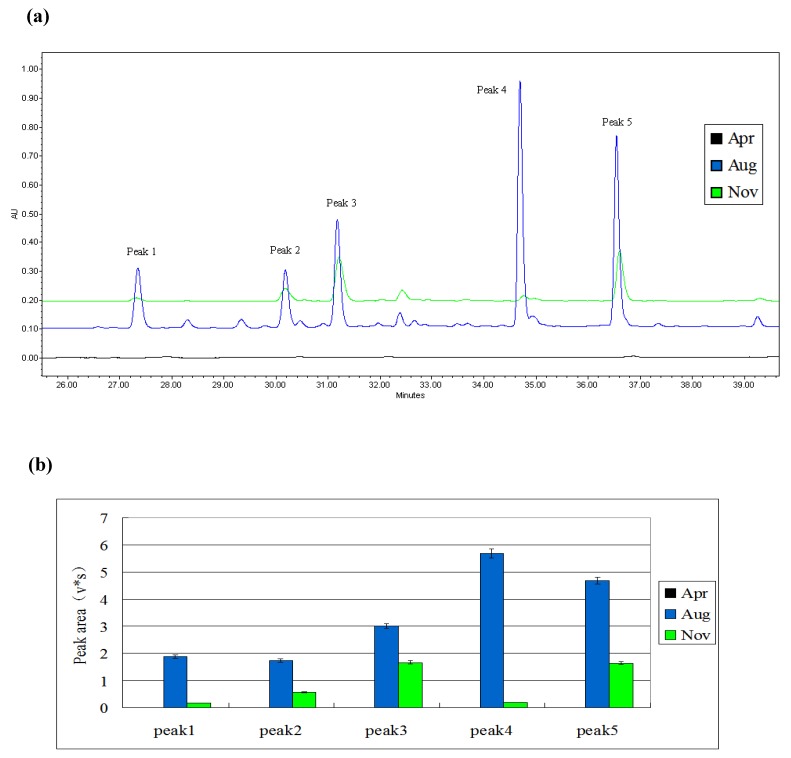
(a) Induced result of different months detected by HPLC fingerprint.HPLC chromatograms of the induced mulberry leaves’ extract obtained in different months. The black curve represents the sample of Apr, the blue curve represents the sample of Aug, and the green curve represents the sample of Nov. A 250 mm Waters Symmetry C18 column was utilized for this experiment. (b) Comparison of peak areas of five induced compounds in different months (Apr/Aug/Nov).

Samples obtained in different months (April, August and November) were irradiated by the same method. The results are shown in Figure 2. Induced peaks appeared in the samples from August and November, but did not appear in those from April. Furthermore, the induced chromatographic peak areas in the samples from August were larger than the peak areas in the samples from November, especially peak 4. Thus, August was selected as the most appropriate inducing month.

#### 2.1.2. Screening of the Length of Induction Time

**Figure 3:**
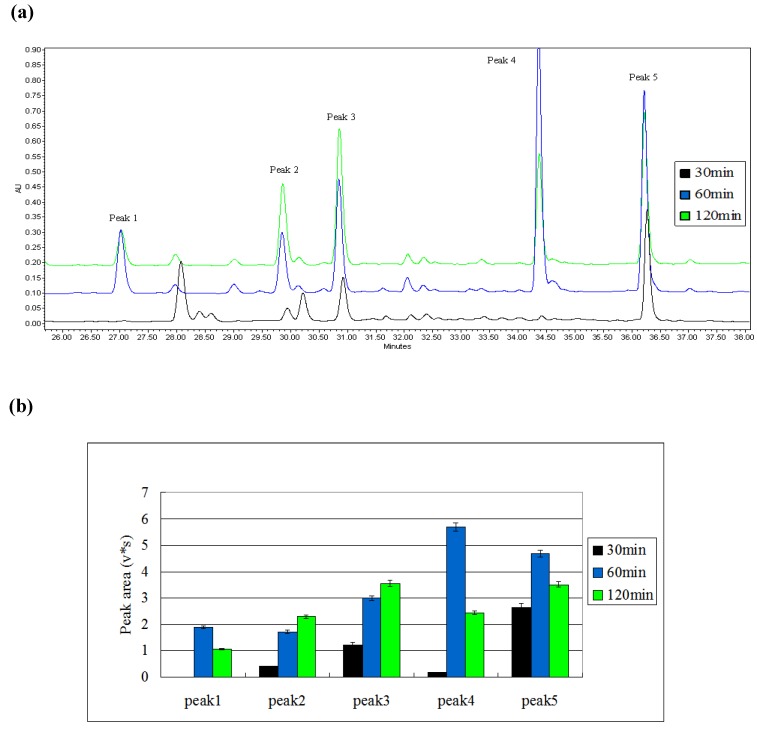
(a) Induced result of different time-period detected by HPLC fingerprint. HPLC chromatograms of the mulberry leaves’ extract induced by different time lengths. The black curve represents the sample of 30min, the blue curve represents the sample of 60 min, and the green curve represents the sample of 120 min. A 250 mm Waters Symmetry C_18 _column was utilized for this experiment. (b) Comparison of peak areas of five induced compounds in different time lengths (Apr/Aug/Nov).

The leaves of August were selected and induced for different durations (30 min, 60 min, and 120 min). The results were given in Figure 3. Content-related peak areas showed that induction for different durations could generate target compounds. As shown, peak 1 and peak 4 were not induced under after 30 min induction, and all five target compounds were induced when the inducing time-period was extended to 60 min. Moreover, the duration of 120 min slightly increased the peak areas of peaks 2 and 3, but significantly reduced the peak areas of peaks 4 and 5 compared with that of 60 min. Thus, 60 min was selected as the most appropriate induction time period.

### 2.2. Induced Compound Identification

Dried, milled plant material of UV-B induction was extracted by maceration and sonicated at room temperature with 100% MeOH. The extract was subjected to separation using silica gel vacuum liquid chromatography (VLC) and gel permeation chromatography. Two monomers of the induced compounds were identified by comparing their NMR data with literature values [17,18]. They were chalcomoracin and moracin N.

### 2.3. Semi- Quantitative Detection

The contents of chalcomoracin and moracin N were semi-quantitively detected by HPLC-UV method for assessing the effects of UV-B induction. The standard curve in 320 nm (the characteristic wavelength of these two compounds) was built. The mulberry leaves were induced in the appropriate inducing conditions *in vitro*, and the result was given in Table 1*.* The content of chalcomoracin was 0.818 mg/g of dry weight, and the content of moracin N was 0.352 mg/g of dry weight. 

**Table 1:** The results of semi-quantitive detection of chalcomoracin and moracin N.

Compound	Regression equation	Test range (μg)	r	Content (mg/10 g)
Chalcomoracin	y = 0.3564x + 0.032	0.2793–2.5137	0.9993	8.18
Moracin N	y = 0.1842x + 0.1912	0.488054– 2.44027	0.9993	3.52

## 3. Discussion

Five significantly changed chromatographic peaks were found in HPLC-DAD fingerprint, and two of them were identified. They were chalcomoracin and moracin N. Unfortunately, peak 1, peak 2, and peak 4 had not been identified. Although peak 4 was higher than peaks 3 and 5, the purified monomer was not obtained. From the UV absorption spectra, the chemical structure of peak 4 is different from other four peaks. One possible explanation was that the chemical corresponding to peak 4 was unstable; and it could be destroyed in the process of separation. The process of separation will be strictly controlled next summer.

According to our UV-B inducing hypothesis, flavonoids are kinds of UV-protective substances. However, the result of the experiment was unexpected; the content of flavonoids was stable after irradiation. Besides, ginkgo leaves, the other sample of our radiation experiment, produced much more flavonoids after induction. Chalcomoracin and moracin N are 2-arylbenzofurans, their UV absorption spectra are similar, incorporating in their chemical structure of phenolic systems; they also have the capacity to protect to the plant of the damage by UV-B radiation. Why was the emergence of 2-arylbenzofurans after enhanced UV-B irradiation? Why mulberry flavonoids were not sensitive to enhanced UV-B irradiation? In the next step, differential proteomics between control and induced samples will be focused in our experiment; changes in enzyme expression could be used to explain the above phenomenon. 

Our results showed that mulberry leaves contain more flavonoids in April than in other months, which was consistent with the existing data [19]. However, in Traditional Chinese Medicine (TCM) theory, the harvest season of mulberry leaves is winter, around October or November. Active ingredients of mulberry leave involve alkaloids, polysaccharide, and phenolic acid. Furthermore, alkaloids or polysaccharide’s signals did not exist in HPLC-DAD fingerprint. Dr. Jiang’s group found that the contents of polysaccharide in mulberry leaves harvested in winter were higher than the ones in other seasons, while the alkaloids varied respectively [20]. Thus, for different active components, harvest season of medical plants can be changed.

Chalcomoracin is a natural Diels-Alder adduct, which was first found in diseased mulberry leaves infected by *Fusarium solani*, and it could not be detected in healthy mulberry leaves. Kokichi Takahashi’s group determined its structure. Furthermore, the compound completely inhibited germination of *Fusarium roseun* and *Biopolaris leersiae* at concentrations of 10–100 µM [17]. In recent years, researchers found that chalcomoracin was distributed in root bark in different species of *Morus* plant, but the content was very low [21]. Chalcomoracin exhibited activity against rhinovirus (MIC: 1.25–2.5 µg/mL) [22]. It also exhibited considerable antimicrobial activity (MIC: 0.78 µg/mL) against methicillin-sensitive *Staphylococcus aureus* (MSSAs, strains FDA 209P and Smith) and methicillin-resistant *Staphylococcus aureus* (MRSAs, strains K3 and ST28). The potency of inhibitory activity of chalcomoracin against these strains was similar to that of vancomycin (MIC:0.39–1.56 µg/mL) [16]. Chalcomoracin was tested for antimicrobial activities against vancomycin-resistant enterococci (VRE). It exhibited considerable antibacterial activity against five VRE strains (VanA-, VanB- and VanC –phenotypes) (MICs: 3.13–6.25 µg/mL) [23]. Chalcomoracin showed moderate cytotoxic activities against five human cancer cell lines, with IC_50_ values ranging from 5.5–7.0 µg/mL, as detected by MTT assay [21]. Recently, sorocenols H, the optical isomer of chalcomoracin, was discovered from *Sorocea muriculata* Roots, and showed significant activity against MRSA with IC_50_ values of 0.5 μM. Furthermore, it displayed antifungal activity against the yeasts *C. neoformans* and *C. albicans*, with an IC_50_ of 5.4 μM each, and the filamentous fungus *A. fumigatus* (IC50:10.0 *μ*M) [24]. Moracin N*,* a plant antitoxin, is a precursor of chalcomoracin, which could be induced by bacteria. Moracin N’s MIC range was 3.125–6.25 µg/mL against gram positive bacteria [25]. 

Chalcomoracin is a Diels-Alder type adduct. Diels-alder type adducts are unique secondary metabolites of *Morus* plants, but they are found in root or stem bark [26]. Similar conclusions have been reported: under normal condition, resveratrol cannot be detected in grapevine leaves, but it exists in pericarp [27,28]. After UV-B irradiation, the compound can be found in leaves. Promoting andgeneralizing this technology, some valuable but rare compounds or their precursors which generally exist in roots or other organs that are hard to be regenerated, could be biosynthesized effectively. There is possibility that more taxinol could be produced by leaves or fruits of *Taxus celebica.*

Furthermore, chalcomoracin is considered to be formed through an enzymatic Diels-Alder (D-A) type reaction of two different isoprenylated phenol. Until 2003, fungal macrophomate synthase, the first natural Diels-Alderase from *Macrophoma commelinae,* had been found [29]. However, no existing documentation has identified the Diels-Alderase of *Morus* [30]. Now, 2D-electrophoresis experiments are under way in our lab to find differentially expressed proteins between normal and mock-treated mulberry leaves. 

Chalcomoracin was first induced by *F. solani.* f. sp. *Mori* [17]. Besides, with *F. solani.* f. sp*. Mori* [25], moracin N could be induced by stem, too*.* According to the compounds generated by induction, to some extent, UV-B inducing and the results of microbial stress test were almost consistent. At present, the possible mechanism of the induction is: greatly enhanced UV-B irradiation forms oxygen free radicals which intend to threat the health of plants and destroy the structure of DNA [15]. As a result, the plant itself produces UV-absorbing substances such as flavonoids and alkaloids through secondary mechanism. The produced metabolites prevent the tissues damage from excessive radiation [31]; furthermore, they repair the injuries caused by oxygen free radicals. In a sense, ultraviolet light acting like a panel point, regulates the whole secondary mechanism. Is microorganism another panel point? Do they reach the same goal by different routes? Especially, the induced compounds represent good antibacterial and anti-virus activity. However, in case of practical applications, UV-B inducing technology is much easier and more efficient than microbial induction. The UV-B inducing content of chalcomoracin was 0.082%, while the microbial induced content was 0.013% [17].

## 4. Experimental Section

### 4.1. Plant Material

The mulberry trees grew in Yuquan campus, Zhejiang University, Hangzhou, China. They were different from previous experiments of plant physiology or environmental chemistry. The samples of the experiment were fresh mulberry leaves *in vitro,* avoiding endangering the survival of plants themselves. 

### 4.2. HPLC Analysis

#### 4.2.1. Sample Preparation

Crushing and screening 1 g UV-B treated air-dry leaves, defatted with chloroform, were extracted three times with 200 mL methanol to give an extract, which was dissolved in 10 mL methanol. The original active ingredients and induced compounds were confirmed in the MeOH solution. The final solution was passed through a 0.22 µm membrane before use. An aliquot of 20 µL of each sample solution was injected into the HPLC for analysis. 

#### 4.2.2. Apparatus and Reagents

HPLC analyses were performed on a Waters 2695 series HPLC system together with column compartment and waters 2998 series photodiode array detector (PDA). HPLC-grade acetonitrile was purchased from Fisher Chemicals (Fair Lawn, NJ, USA). NMR spectra were recorded on a Bruker 400 spectrometer. HPLC-grade phosphoric acid was purchased from Tedia Company, Inc. (Fairfield, OH, USA). The water used in the experiment was doubly distilled in the laboratory. Other chemicals and solvents were of analytical grade.

#### 4.2.3. Fingerprint Chromatographic Condition of before/after Induced Mulberry Leaves

The HPLC fingerprinting analysis was carried out on a Waters Symmetry C_18_ column (250mm×4.6 mmI.D, 5 µm). A binary gradient elution system, which was composed of acetonitrile as solvent A and 0.1% phosphoric acid in water as solvent B, was applied for the fingerprint analysis with the gradient elution as follows: 0–25 min, 10–50% A; 25–40 min, 50–95% A; 40–45 min, 95% A; 45.01–52 min, 10% A. The flow rate of mobile phase was 1 mL/min, and column temperature was maintained at 40 °C. The PDA detector was set at 320 nm, and the on-line UV spectra were recorded in the range of 210–400 nm.

### 4.3. Induce Condition Screening

The peak areas of five significantly changed chromatographic peaks were selected as screening indicators.

#### 4.3.1. Month

Yangtze River Delta is the main location of mulberry leaves. Their growing period is from March to November. The inducing sensitivity varied with months. Therefore, samples of April, August and November were chosen in our experiment. 

#### 4.3.2. Intensity of UV-B Light

Previous data of experiments showed that, low-dose (15w) and long-term UV-B induction is effective for plant *in vivo*. Nevertheless, our assumption was based on the results obtained in the experiments with leaves *in vitro*, so high-power UV-B source and short-term induction was selected. The UV-B inducing device was made of a glass house (1.5m*1m*1.2m) and 3 tubular low-pressure mercury-vapor lamps (TL40w/12RS, 280–320 nm, Philips) emitting approximately 40w of UV-B irradiation at 306 nm. The lamp was fixed in the upper side of the device, and the distance of irradiation was 20cm. Besides, in order to prevent the damage of O_3_ [31], a ventilation device was fixed in induction box.

#### 4.3.3. Culture Condition

Long-term culture was not conducive to leaves *in vitro*, and visible light could repair injury generated by UV light [32]. As a result, the selected culture condition was: 24 hours in the dark, 30 °C, with 100% humidity. 

### 4.4. Extraction and Isolation

Dried, milled plant material of UV-B induction (1000 g), was extracted by maceration and sonicated at room temperature with 100% MeOH. The extract was evaporated in vacuum to yield 135.5 g of a dried residue. All of the extract was subjected to separation using silica gel vacuum liquid chromatography (VLC), which was eluted with chloroform and gradient chloroform /MeOH to 20% MeOH. Five fractions (A to E) were collected. Fraction B (1200 mg) was further chromatographed on Sephadex LH-20 using chloroform/MeOH (1/1, v/v) as eluent to yield 1 (2.9 mg). Fractions C (500 mg) were passed over Sephadex LH-20 eluted with chloroform-MeOH (1:1), followed by final purification by C_18_-HPLC using gradient MeOH/H2O (80/20, v/v) to 100% MeOH to yield **2 **(8.3 mg).

### 4.5. Compounds Analysis

#### 4.5.1. Chalcomoracin

**Figure 4:**
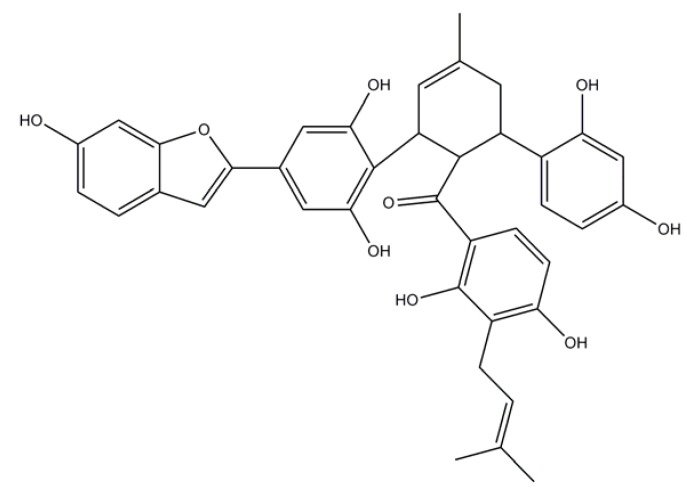
Chemical structure of chalcomoracin identified in induced mulberry leaves (Peak 5).

Peak 5, Figure 4. Yellowish powder; FABMS *m/z*: 649[M+H]^ +^_._^1^H-NMR (500 MHz, methanol *d*_4_): *δ*_H_ 8.42 (1H, *d*, *J*= 9.3Hz, H-14"), 7.34 (1H, *d*, *J*= 8.1Hz, H-4), 7.00 (1H, *d*, *J* = 8.0Hz, H-20"), 6.92 (2H, *br*. *s*, H-3,7), 6.77 (1H, *m*, H-5), 6.76 (2H, *br*. *s*, H-2', 6'), 6.50 (1H, *d*, *J*=2.l Hz, H-17"), 6.42 (1H, *d*, *J* = 9.3 Hz, H-13 "), 6.32 (1H, *dd*, *J* = 8.0,2.1Hz, H-19"), 5.77 (1H, *br. s*, H-2"), 5.16 (1H, *t*, *J*=6.9 Hz, H-22"), 4.63 (1H, *t*, *J* = 4.5Hz, H-4"), 4.11 (1H, *br. s*, H-3 "), 3.75 (1 H, *t*, *J* = 4.5 Hz, H-5"), 3.25 (2H, *d*, *J* = 6.9Hz, H-21"), 2.52 (1 H, m, H-6"), 2.21 (1H, *m*, H-6"), 1.93 (3H, *s*, H-7"), 1.70 (3H, *s*, H-24"), 1.56 (3H, *s*, H-25"). ^13^C-NMR (125 MHz, methanol *d_4_*): *δ*_C_ 209.7 (C-8"), 164.6(C-10"), 163.3 (C-12"), 157.8 (C-6, 18"), 156.6 (C-16"), 156.5 (C-3', 5'), 156.3 (C-2),155.4 (C-7α), 133.8 (C-1”),132.1 (C-14"), 131.5 (C-23"), 130.9 (C-1'), 128.7 (C-20"),124.4 (C-2"), 123.1 (C-22"), 122.6 (C-3a), 121.8 (C-4, 15"), 116.6 (C-11"), 115.8(C-4'), 113.4 (C-9"), 113.0 (C-5), 108.1 (C-13"), 107.4 (C-19"), 104.8 (C-2', 6'), 103.4 (C-17"), 101.8 (C-3), 98.3 (C-7), 47.8 (C-4"), 36.5 (C-5"), 33.1 (C-3"), 32.2(C-6"), 25.8 (C-24"), 23.8 (C-7"), 22.2 (C-21"), 17.8 (C-25").

#### 4.5.2. Moracin N

**Figure 5:**
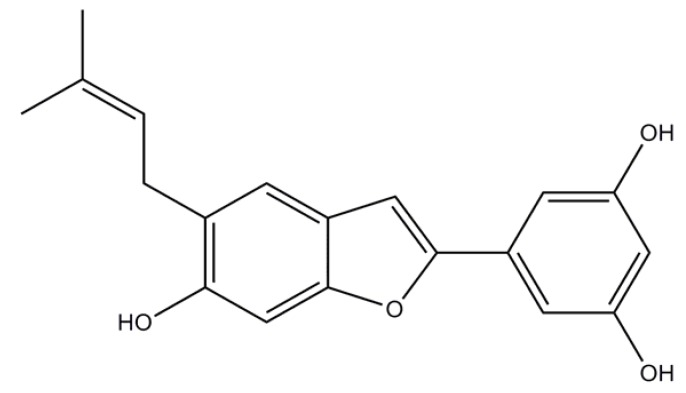
Chemical structure of moracin N identified in induced mulberry leaves (Peak 3).

Peak 3, *figure 5*.Yellowish powder; 1HNMR (500 MHz, methanol *d_4_*): *δ*_H_ 7.09 (1H, *s*, H-4), 6.79 (1H, *s*, H-7), 6.76 (1H, *s*, H-3), 6.65 (1H, *s*, H-2′), 6.64 (1H, *s*, H-6′), 6.13 (1H, *t*, *J*= 4.3, 2.2 Hz, H-4′), 5.26 (1H, *t*, *J*= 2.8, 1.4 Hz, H-9), 3.25 (2H, *m*, H-8), 1.65 (3H, *s*, H-11), 1.63 (3H, *s*, H-12); ^13^C-NMR (125 MHz, methanol *d_4_*): *δ*_C _18.2 (C-11), 26.4 (C-12), 29.9 (C-8), 98.3 (C-7), 102.7 (C-3), 103.8 (C-4′), 104.3 (C-2′, C-6′), 121.8 (C-4), 123.2 (C-4a), 124.8 (C-8), 126.6 (C-6), 133.3 (C-10), 134.4 (C-1′), 155.0 (C-6), 155.9 (C-7a), 156.2 (C-2), 1660.3 (C-3′, C-5′). 

### 4.6. Semi-Quantitative Analysis of Inducing Result

Methanol stock solutions containing reference compounds were prepared and diluted to appropriate concentrations for the construction of calibration curves. At least five concentrations of the solution were analyzed in duplicates, and then the calibration curves were constructed by plotting the peak areas versus the concentration of each analyte. The results are shown in Table 1. The high correlation coefficient values (*r* > 0.999) indicated good linearity between their peak areas (*y*) and the investigated compound concentration (*x*, μg) in relatively wide concentration ranges.

## 5. Conclusions

According to our results, the UV-B radiation method could induce chalcomoracin and moracin N in mulberry leaves *in vitro*. The contents of induced chalcomoracin and moracin N were 8.18 mg/10 g of dry weight and 3.52 mg/10 g of dry weight.
